# Neonatal sequential organ failure assessment score within 72 h after delivery reliably predicts bronchopulmonary dysplasia in very preterm infants

**DOI:** 10.3389/fped.2023.1233189

**Published:** 2023-09-29

**Authors:** Doudou Xu, Ziwei Dong, Xiaoli Yin, Yuanyuan Yang, Yang Wang

**Affiliations:** Department of Pediatrics, The First Affiliated Hospital of Anhui Medical University, Hefei, China

**Keywords:** neonatal sequential organ failure assessment, premature infants, bronchopulmonary dysplasia, neonatal intensive care unit, predict

## Abstract

**Background:**

The neonatal sequential organ failure assessment (nSOFA) score is an operational definition of organ dysfunction employed to predict sepsis-associated mortality. However, the relationship between the nSOFA score and bronchopulmonary dysplasia (BPD) has not been investigated clearly. This study evaluates whether the nSOFA score within 72 h after delivery could be used to predict the occurrence of BPD in very preterm infants.

**Methods:**

In this retrospective, single-center cohort study, preterm infants born between 2019 and 2021 were investigated, the nSOFA score was calculated from medical records after admission to the neonatal intensive care unit (NICU) within 72 h after delivery, and the peak value was used for calculation. A logistic regression model was used to evaluate the relationship between the nSOFA score and BPD. Propensity score matching and subgroup analysis were performed to verify the reliability of the results.

**Results:**

Of 238 infants meeting the inclusion criteria, 93 infants (39.1%) were diagnosed with BPD. The receiver operating characteristic curve of the nSOFA score in predicting BPD was 0.790 [95% confidence interval (CI): 0.731–0.849]. The logistic regression model showed that an increment of one in the nSOFA score was related to a 2.09-fold increase in the odds of BPD (95% CI: 1.57–2.76) and 6.36-fold increase when the nSOFA score was higher than 1.5 (95% CI: 2.73–14.79).

**Conclusions:**

The nSOFA score within 72 h after delivery is independently related to BPD and can be used to identify high-risk infants and implement early interventions.

## Introduction

Although the survival rate of extremely preterm infants has improved over the past years, the incidence of bronchopulmonary dysplasia (BPD) remains unchanged or even is increasing (1, 2). Studies have reported that the global incidence rate of BPD in extremely preterm infants ranges from 10% to 89%, emphasizing the important health implications of BPD (3).

BPD is a multifactorial chronic respiratory illness with increased mortality and morbidity, including BPD-associated pulmonary hypertension (4, 5), neurodevelopmental deficits (6, 7), wheezing and asthma (8), lung dysfunction throughout childhood and adolescence (9, 10), and susceptibility to chronic obstructive pulmonary disease in adulthood (11, 12). These pose a considerable burden to the affected infants, their families, and the public health system (13). Therefore, identifying infants at high risk for BPD is important for the development of preventive strategies.

The neonatal sequential organ failure assessment (nSOFA) score was developed to predict mortality in infants with late-onset infection (14, 15). Recent studies have shown that the nSOFA score is independently associated with the mortality of neonates with respiratory distress syndrome (RDS) (16) and necrotizing enterocolitis (NEC) (17). However, the relationship between nSOFA scores and BPD is unclear.

This study evaluates whether the nSOFA score within 72 h after delivery could predict the occurrence of BPD in very preterm neonates. Our finding may broaden the clinical application of the nSOFA score and help clinicians quickly identify infants at high risk for BPD and implement early interventions.

## Methods

### Study design and population

This study was a retrospective, single-center cohort study of preterm infants admitted to the neonatal intensive care unit (NICU) in the First Affiliated Hospital of Anhui Medical University between 1 July 2019 and 31 March 2021. All infants were born in the Obstetrics Department of the same hospital and transferred to NICU immediately after birth or recovery. Other inclusion criteria were as follows: (1) gestational age (GA) was less than 32 weeks, (2) birth weight (BW) was lower than 1,500 g, and (3) preterm infants had a survival time ≥28 days. The exclusion criteria were as follows: (1) infants had a congenital malformation of vital organs or genetic/metabolic diseases, (2) infants had culture-proven sepsis, (3) infants had stage IIA or higher NEC, and (4) the clinical information was incomplete. The Ethics and Research Committees of the First Affiliated Hospital of Anhui Medical University approved this study (Quick-PJ 2021-05-33).

### Data collection

Information on the demographics, perinatal characteristics, treatments during hospitalization, and complications after birth was collected from medical records. The demographics included GA, BW, gender, assisted reproduction, multiple births, cesarean delivery, maternal age, and score for clinical risk index for babies (CRIB)-II. The perinatal characteristics included pregnancy complications (gestational diabetes mellitus, hypertensive disorders of pregnancy, prolonged rupture of membranes, antenatal steroids, 5 min Apgar score, and intubation in the delivery room). Treatments during hospitalization included pulmonary surfactant, caffeine, and duration of mechanical ventilation. Complications after birth included BPD, RDS, and patent ductus arteriosus. Clinical characteristics required to calculate the nSOFA score included the peripheral oxygen saturation; fraction of inspired oxygen to achieve the peripheral oxygen saturation; intubation and mechanical ventilation; any requirement for glucocorticoid, inotropic, or vasoactive drugs; and the latest platelet count.

### Application of nSOFA score

The nSOFA score was calculated after admission to the NICU within the first 72 h after delivery, and the peak value was used for the calculation. The nSOFA system uses categorical scores with a total score ranging from 0 as best to 15 as worst, which were used to objectively describe dynamic changes in respiratory deterioration (0–8), hemodynamic compromise (0–4), and hematologic dysfunction (0–3) (15) (Table 1).

**Table 1:** Neonatal sequential organ failure assessment (nSOFA) components and scoring^a^.

Component	nSOFA score				
Respiratory score	0	2	4	6	8
Criteria	Not intubated or intubated, SpO_2_/FiO_2_≥ 300	Intubated, SpO_2_/FiO_2_ <300	Intubated, SpO_2_/FiO_2_ <200	Intubated, SpO_2_/FiO_2_ <150	Intubated, SpO_2_/FiO_2_ <100
Cardiovascular score	0	1	2	3	4
Criteria^b^	No inotropes and no systemic corticosteroid treatment	No inotropes and systemic corticosteroid treatment	1 inotrope and no systemic corticosteroid treatment	≥2 inotropes or 1 inotrope and systemic corticosteroid treatment	≥2 inotropes and systemic corticosteroid treatment
Hematologic score	0	1	2	3	NA
Criteria^c^	Platelet count^d^ ≥150 × 10^3^	Platelet count 100–149 × 10^3^	Platelet count <100 × 10^3^	Platelet count <50 × 10^3^	

nSOFA, neonatal sequential organ failure assessment; SpO_2_, peripheral oximetric saturation; FiO_2_, fraction of inspiratory oxygen; NA, not applicable.

^a^ Score range, 0 (best) to 15 (worst).

^b^ Medications included inotropic or vasoactive including dopamine, dobutamine, epinephrine, norepinephrine, vasopressin, and phenylephrine.

^c^ The latest platelet count available.

^d^ SI conversion factor: to convert platelet count to ×10^9^/L, multiply by 1.

### Outcome

The study outcome was the presence or absence of BPD. BPD was determined according to the National Institutes of Health consensus definition (18) and defined as required supplemental oxygen for ≥28 days.

### Circulatory support

Circulatory support was based on the potential pathophysiology of circulatory system damage, mean blood pressure, laboratory, clinical signs of hypoperfusion, and bedside echocardiography. Dopamine was the most common positive inotropic drug. Dobutamine was the most popular second-line treatment when the initial inotrope therapy failed. If the above treatment was ineffective, adrenaline or norepinephrine was used as the third-line treatment. When refractive hypotension or severe blood loss/asphyxia was present and affected adrenal perfusion, corticosteroids were used for the treatment (19, 20).

### Propensity score matching

Given that infants were not randomly assigned to receive antibiotics, propensity scoring was used to control for potential confounding. BPD cases were matched with controls at 1:1 based on GA, BW, gender, assisted reproduction, multiple births, cesarean delivery, maternal age, and CRIB-II score. Patients were matched on the logit of propensity score using optimal matching with a caliper width of 0.02. Matching was performed without replacement.

### Statistical analyses

Descriptive statistics were calculated for demographics and clinical characteristics between the original and matched populations. Values are presented as medians and interquartile ranges for continuous variables and as numbers and percentages for categorical variables. The continuous variables were compared using the Mann–Whitney test, and the categorical variables using the Chi-squared test.

The accuracy of the total nSOFA and component nSOFA scores in predicting BPD was calculated by the area under the receiver operating characteristic curve (AUROC). The cutoff value of the receiver operating characteristic (ROC) curve was determined by calculating the Youden index, which divided the population into the high nSOFA and low nSOFA groups.

Logistic regression models were used to evaluate the relationship between the nSOFA score and BPD in the original and matched populations. Model 1 was unadjusted, model 2 was adjusted for GA and BW, and model 3 was adjusted for GA, BW, CRIB-II score, 5 min Apgar score, intubation in the delivery room, and mechanical ventilation >7 days. A subgroup analysis was performed to examine the relationship after modifying for gender, mode of delivery, and conception.

Statistical analyses were performed using the statistical software packages R 3.3.2 (http://www.R-project.org, The R Foundation) and Free Statistics software version 1.4 (21). A two-sided *P*-value of <0.05 was considered statistically significant.

## Results

### Baseline characteristics

**Figure 1:**
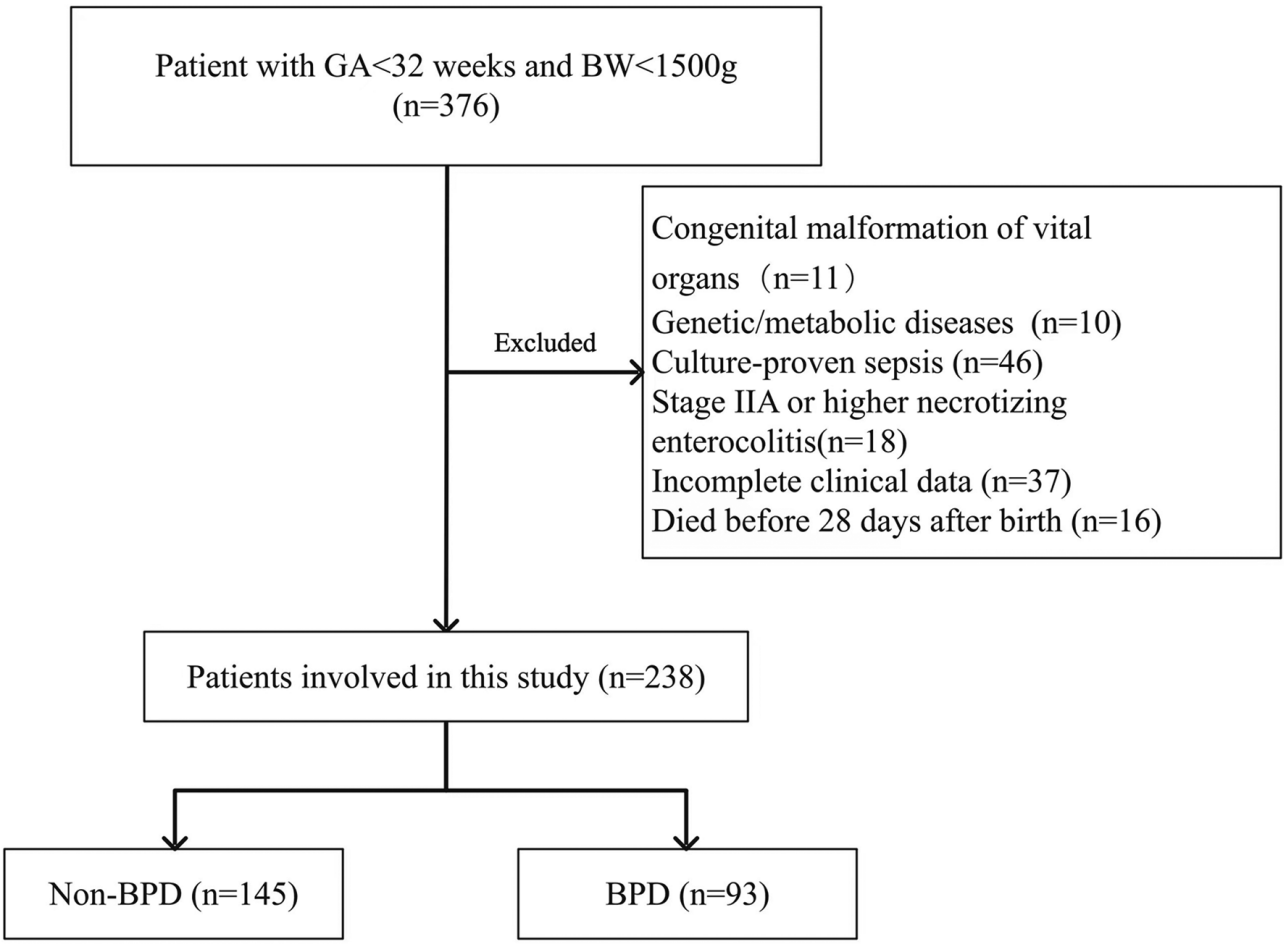
Flow diagram of patient selection.

During the study period, 376 premature infants had GA less than 32 weeks and BW less than 1,500 g. Infants with congenital malformation of vital organs (*n* = 11), genetic or metabolic diseases (*n* = 10), and culture-proven sepsis (*n* = 46) were excluded. In addition, infants who had stage IIA or higher NEC (*n* = 18), had incomplete clinical data (*n* = 37), and died before 28 days after birth (*n* = 16) were also excluded. The remaining 238 infants were included in our analysis, of which 93 were diagnosed with BPD and 145 were free of BPD. Figure 1 shows the study flowchart. Before propensity score matching, compared with the low nSOFA group, the GA was smaller, the BW was lower, and the CRIB-II score was higher in infants with high nSOFA scores (all *P *< 0.001). After propensity score matching, there were no significant differences in the GA, BW, gender, multiple births, cesarean delivery, assisted reproduction, CRIB-II score, and maternal age (Table 2).

**Table 2:** Baseline characteristics of patients.

Characteristics	Total patients (*n* = 238)	Before propensity score matching		*P*-value	After propensity score matching		*P-*value
		Low nSOFA (*n* = 138)	High nSOFA (*n* = 100)		Low nSOFA (*n* = 63)	High nSOFA (*n* = 63)	
GA (week), median (IQR)	29.4 (28.4, 30.6)	29.9 (29.0, 30.7)	28.9 (28.1, 30.0)	<0.001	29.3 (28.7, 30.4)	29.6 (28.4, 30.3)	0.723
BW (g), median (IQR)	1,190.0 (1,005.0, 1,310.0)	1,250.0 (1,105.0, 1,347.5)	1,075.0 (970.0, 1,200.0)	<0.001	1,175.0 (995.0, 1,300.0)	1,170.0 (1,030.0, 1,255.0)	0.798
CRIB-II score, median (IQR)	7.0 (5.0, 9.0)	6.0 (5.0, 8.0)	8.0 (7.0, 10.0)	<0.001	7.0 (6.0, 8.0)	7.0 (6.0, 8.0)	0.963
Male sex, no. (%)	130 (54.6)	75 (54.3)	55 (55)	0.921	32 (50.8)	31 (49.2)	0.859
Multiple births, no. (%)	81 (34.0)	48 (34.8)	33 (33)	0.775	18 (28.6)	19 (30.2)	0.845
Cesarean delivery, no. (%)	161 (67.6)	91 (65.9)	70 (70)	0.509	45 (71.4)	44 (69.8)	0.845
Assisted reproduction, no. (%)	65 (27.3)	39 (28.3)	26 (26)	0.699	15 (23.8)	18 (28.6)	0.543
Maternal age (year), median (IQR)	30.0 (27.0, 33.0)	30.0 (27.0, 33.0)	30.5 (27.0, 34.0)	0.692	30.0 (27.0, 33.0)	30.0 (26.0, 33.0)	0.586
HDP, no. (%)	73 (30.7)	40 (29)	33 (33)	0.507	21 (33.3)	22 (34.9)	0.851
GDM, no. (%)	40 (16.8)	21 (15.2)	19 (19)	0.441	9 (14.3)	15 (23.8)	0.173
PROM >18 h, no. (%)	63 (26.5)	38 (27.5)	25 (25)	0.662	17 (27)	18 (28.6)	0.842
Antenatal steroids, no. (%)	198 (83.2)	113 (81.9)	85 (85)	0.526	56 (88.9)	55 (87.3)	0.783
5 min Apgar score, median (IQR)	8.0 (7.0, 9.0)	9.0 (8.0, 9.0)	8.0 (7.0, 8.0)	<0.001	9.0 (8.0, 9.0)	8.0 (7.0, 8.0)	0.007
Intubated in the delivery room, no. (%)	88 (37.0)	26 (18.8)	62 (62)	<0.001	14 (22.2)	37 (58.7)	<0.001
PS, no. (%)	190 (79.8)	99 (71.7)	91 (91)	<0.001	48 (76.2)	56 (88.9)	0.06
Caffeine, no. (%)	191 (80.3)	107 (77.5)	84 (84)	0.216	55 (87.3)	50 (79.4)	0.232
Mechanical ventilation time>7 days, no. (%)	66 (27.7)	13 (9.4)	53 (53)	<0.001	6 (9.5)	32 (50.8)	<0.001
BPD, no. (%)	93 (39.1)	28 (20.3)	65 (65)	<0.001	18 (28.6)	38 (60.3)	<0.001
RDS, no. (%)	224 (94.1)	125 (90.6)	99 (99.0)	0.006	60 (95.2)	62 (98.4)	0.619
PDA, no. (%)	49 (20.6)	20 (14.5)	29 (29)	0.006	10 (15.9)	15 (23.8)	0.264
nSOFA score	1.0 (0.0, 3.0)	0.0 (0.0, 0.0)	3.0 (2.0, 5.0)	<0.001	0.0 (0.0, 1.0)	3.0 (2.0, 4.0)	<0.001

nSOFA, neonatal sequential organ failure assessment; GA, gestational age; IQR, interquartile range; BW, birth weight; CRIB-II, clinical Risk Index in Babies-II; HDP, hypertensive disorders of pregnancy; GDM, gestational diabetes mellitus; PROM, prolonged rupture of membranes; PS, pulmonary surfactant; BPD, bronchopulmonary dysplasia; RDS, respiratory distress syndrome; PDA, patent ductus arteriosus.

### nSOFA score utility for BPD

**Figure 2:**
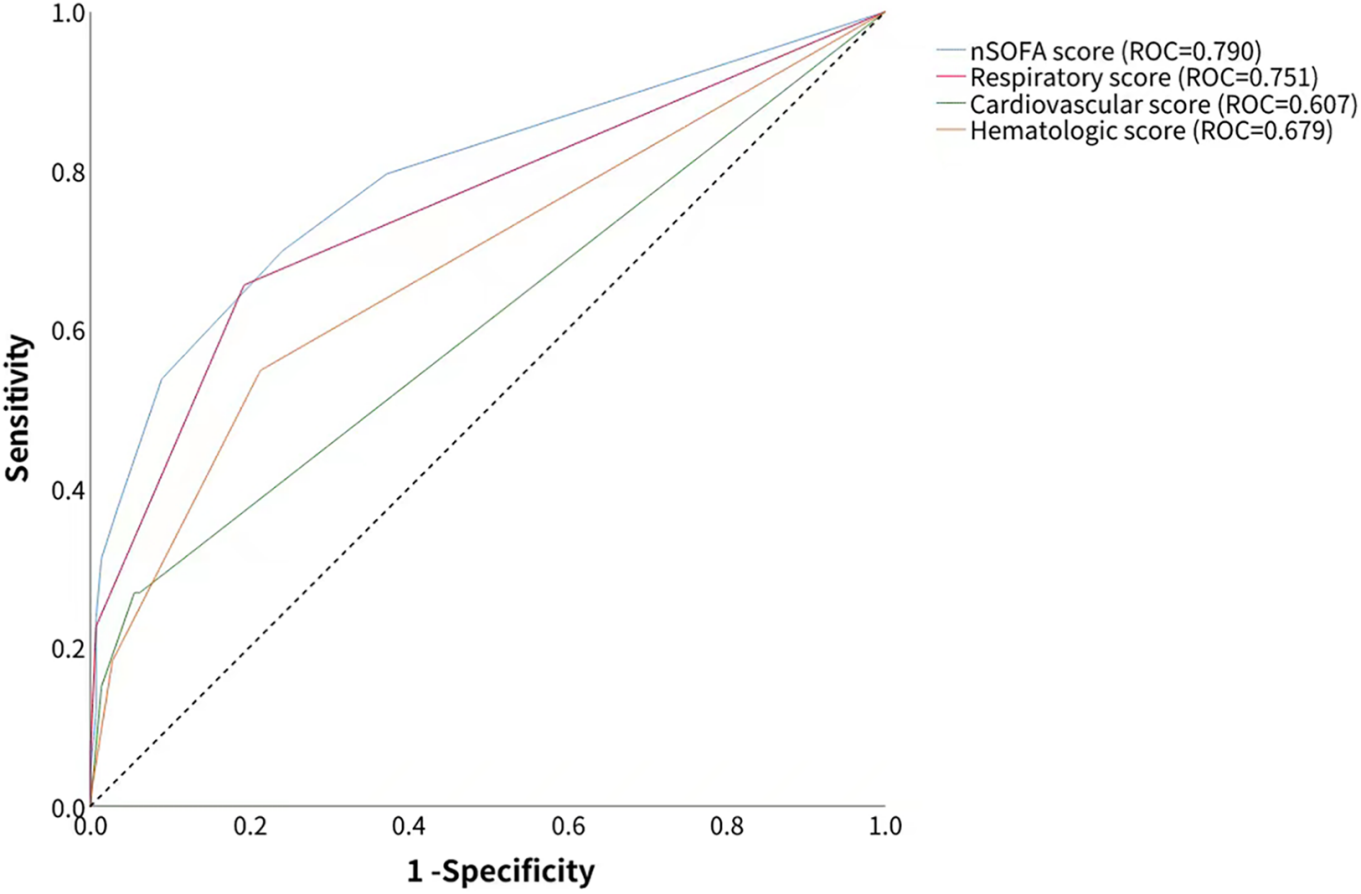
Accuracy of total nSOFA score and component nSOFA score in predicting BPD was calculated by the receiver operating characteristic curve.

The AUROC of the nSOFA score in predicting BPD was 0.790 [95% confidence interval (CI): 0.731–0.849], which was higher than the predictive efficacy of the component score: the AUROC of the respiratory score was 0.751 (95% CI: 0.693–0.809), the AUROC of the cardiovascular score was 0.607 (95% CI: 0.557–0.656), and the AUROC of the hematologic score was 0.679 (95% CI: 0.618–0.741) (Figure 2). The optimal cutoff value of the nSOFA score for BPD in preterm infants was 1.5, which divided the population into the high nSOFA (*n* = 100) and low nSOFA (*n* = 138) groups.

### Association between nSOFA score and BPD

A regression analysis was performed to identify factors associated with BPD. In the unadjusted model, the nSOFA score was significantly associated with the risk of BPD (OR = 1.9, 95% CI: 1.57–2.3). In the multivariate regression model, after adjusting for GA and BW, the odds ratio was 1.74 (95% CI: 1.43–2.12). After adjusting for GA, BW, CRIB-II score, 5 min Apgar score, intubation in the delivery room, and mechanical ventilation >7 days, the odds ratio was 2.09 (95% CI: 1.57–2.76). In addition, the high nSOFA score significantly increased the risk of BPD as compared with the low nSOFA score (aOR = 6.36, 95% CI: 2.73–14.79) (Table 3). Propensity score matching showed that the relationship between the nSOFA score and BPD remained stable (Table 3).

**Table 3:** Relationship between nSOFA score and BPD.

Variable	Event	Model 1		Model 2		Model 3	
		OR (95% CI)	*P*-value	OR (95% CI)	*P*-value	OR (95% CI)	*P*-value
Original cohort							
nSOFA	93/238	1.9 (1.57–2.3)	<0.001	1.74 (1.43–2.12)	<0.001	2.09 (1.57–2.76)	<0.001
nSOFA group							
Low nSOFA	138/238	Reference		Reference		Reference	
High nSOFA	100/238	7.3 (4.07–13.08)	<0.001	5.19 (2.75–9.78)	<0.001	6.36 (2.73–14.79)	<0.001
Matched cohort							
nSOFA	56/126	1.77 (1.36–2.3)	<0.001	1.85 (1.4–2.44)	<0.001	2.07 (1.44–2.99)	<0.001
nSOFA group							
Low nSOFA	18/63	Reference		Reference		Reference	
High nSOFA	38/63	3.8 (1.81–8)	<0.001	4.07 (1.85–8.96)	<0.001	4.53 (1.7–12.06)	0.002

nSOFA, neonatal sequential organ failure assessment; BPD, bronchopulmonary dysplasia; CI, confidence interval. Model 1, unadjusted. Model 2, adjusted for gestational age and birth weight. Model 3, adjusted for gestational age, birth weight, CRIB-II score, 5 min Apgar score, intubation in delivery room, and mechanical ventilation >7 days.

### Sensitivity analysis

**Figure 3:**
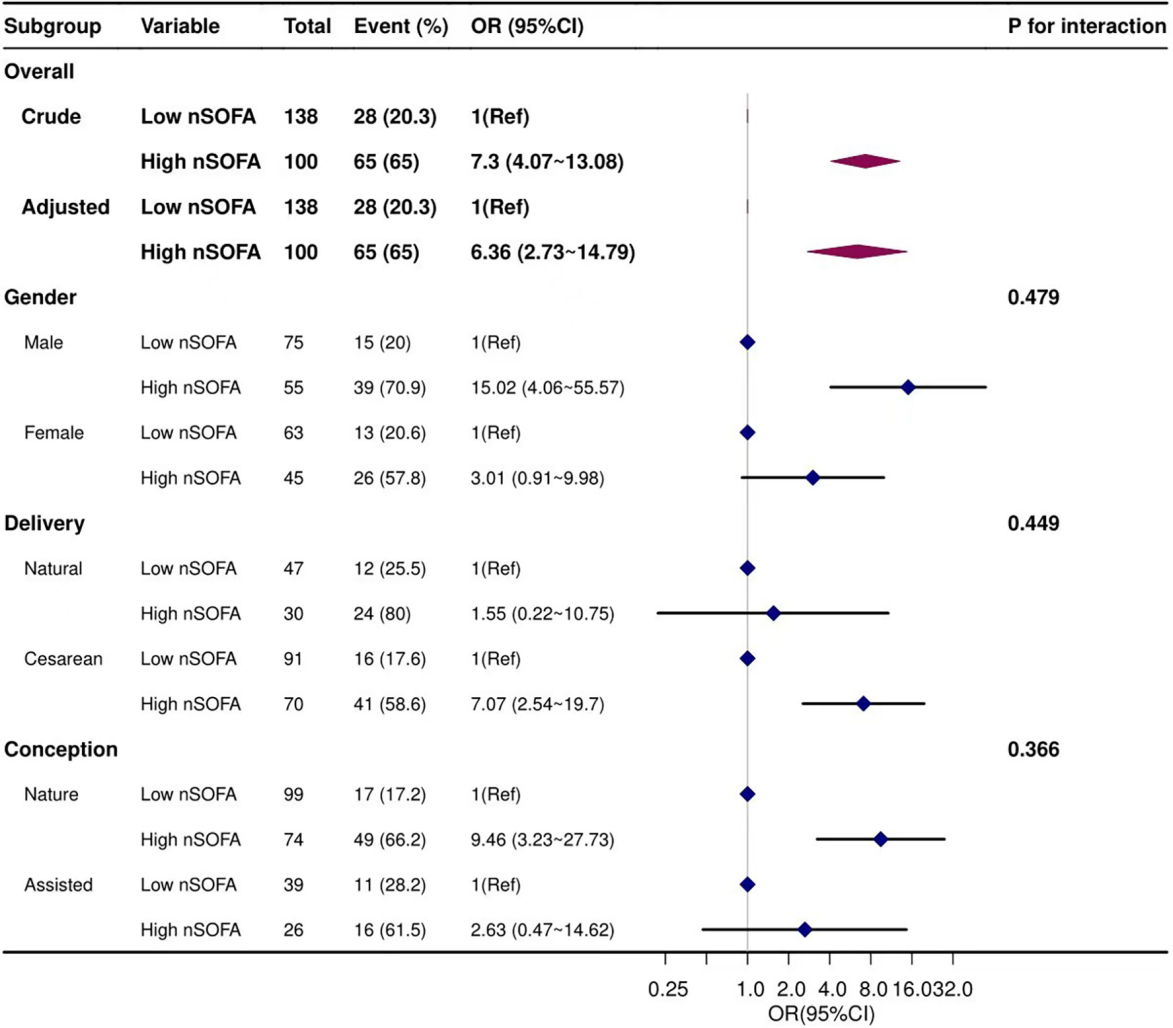
Forest plots of subgroup analysis. Adjusted for gestational age, birth weight, CRIB-II score, 5 min Apgar score, intubation in the delivery room, and mechanical ventilation>7 day.

Stratified analyses were performed to evaluate whether the association between the nSOFA score and BPD varied by gender, mode of delivery, and conception. Results showed that none of the subgroups significantly affected this association (Figure 3).

## Discussion

To date, few studies have investigated the relationship between the nSOFA score and BPD in very preterm infants. In this retrospective study, our results showed that the nSOFA score was independently associated with BPD. An increment of one in the nSOFA score was related to a 90% increase in the odds of BPD and 7.3-fold increase when the nSOFA score was higher than 1.5. The relationship remained stable even after adjusting for other confounding factors. These findings indicate that the nSOFA score may become a useful indicator for the early prediction of BPD in very preterm infants.

In 2016, the consensus panel members of the European Society of Intensive Care Medicine and the Society of Critical Care Medicine proposed the SOFA score for the standardized assessment of organ dysfunction (22). Matics et al. (23) validated a pediatric version of the SOFA score (pSOFA) in critically ill children with age-adjusted variables. The SOFA and pSOFA scores have been commonly used in adult and pediatric patients to predict sepsis-related mortality (24, 25). Wynn and Polin (26) employed the nSOFA to predict mortality of preterm infants with very low BW with late-onset sepsis (LOS).

Several centers have confirmed the predictive value of the nSOFA score, showing its generalizability. In a multicenter retrospective cohort study on 653 preterm infants with very low BW with late-onset infection (bacteremia, intestinal perforation, or fungemia), the nSOFA score in predicting mortality has an AUROC of 0.81 during sepsis evaluation (95% CI: 0.76–0.85), 0.87 (95% CI: 0.83–0.92) at 6 h postsepsis evaluation, and 0.86 (95% CI: 0.81–0.91) at 12 h postsepsis evaluation (15). Analyses stratified by gender, GA, or pathogen class did not compromise the association of the nSOFA score and mortality. Another study involving 956 very low BW infants with GA <33 weeks showed that the nSOFA score at 12 h after the time of blood culture has an AUROC of 0.91 (95% CI: 0.84–0.99) in predicting mortality during LOS or NEC (27). Our results were consistent with the above findings that the nSOFA score within 72 h after delivery had an AUROC of 0.790 (95% CI: 0.731–0.849) in predicting BPD. Therefore, several centers have integrated the nSOFA score into electronic health records of NICU infants for prospective studies and quality improvement (15).

Our study also revealed that the respiratory score was the strongest predictor of BPD in very preterm infants with an AUROC of 0.751 (95% CI: 0.693–0.809), which was nearly the same as the predictive performance of the whole nSOFA scale (AUROC = 0.790). This suggests that BPD is secondary to the influence of genetic (28) and environmental interactions (such as hyperoxia and invasive mechanical ventilation) (29) on the immature lung that causes alveolar hypoplasia and pulmonary vascular malformations. As we all know, according to the 2018 criteria from the National Institute for Child Health and Human Development (30), BPD is diagnosed based on the need for respiratory support for ≥3 days to maintain oxygen saturation at 90%–95% at 36 weeks postmenstrual age. The respiratory subscale of the nSOFA score includes both invasive ventilatory support and oxygen requirements to maintain normal peripheral saturation. However, hypotension and thrombocytopenia may be more suggestive of sepsis assessment, which means that if a premature infant is not intubated in the first 72 h, the risk of BPD is dramatically lower. Some strategies can be used to achieve this goal in the first days of life, such as continuous positive airway pressure in the delivery room, early selective use of surfactant, and administration of less invasive surfactant.

Identifying the risk for BPD in the early stage is important to assist healthcare providers in providing more targeted interventions. Therefore, some models have been established to predict BPD and its severity in recent years. Most prediction models employ clinical indicators, imaging findings, complicated biomarkers, and even genomic parameters as factors (31, 32), aiming to identify infants at high risk of BPD based on traditional statistics or artificial intelligence technology (33, 34). It is hard to generalize these models because of the potential difficulties and complexities in assessing these variables in clinical practice (35) or the increased medical cost (34). Thus, the early identiﬁcation of infants with high risk for BPD remains a significant challenge in clinical practice. The nSOFA score is easy to obtain, applicable early after delivery, and reliable in predicting BPD. These features of the nSOFA score have not been reported in previous prediction models and also indicate that it can be used in most hospitals.

There were several limitations in this study. First, this was a single-center retrospective study with a small sample size. Thus, bias was unavoidable, although propensity score matching was conducted to reduce confounders. In the future, more multicenter prospective cohort studies with large sample sizes are warranted to further explore the association between the nSOFA score and risk of BPD in preterm infants. Second, some factors that might be related to BPD were not included in this study, such as early fluid management and chorioamnionitis. Third, the BPD in this study was mainly based on oxygen supplementation ≥28 days. Since respiratory support at 36 weeks postmenstrual age was unknown, classification according to the new criteria for BPD could not be achieved. The relationship between the nSOFA score and BPD based on the new BPD classification criteria should be further explored in more clinical studies.

## Conclusion

In this study, our results indicate that an elevated nSOFA score within 72 h after delivery is associated with an increased risk of BPD in very preterm infants. The nSOFA score may be used to facilitate the precise classification of patients and implement targeted interventions in the early stage of BPD.

## Data Availability

The original contributions presented in the study are included in the article/Supplementary Material, further inquiries can be directed to the corresponding author.
